# Influence of age on sperm characteristics evaluated by light and electron microscopies

**DOI:** 10.1038/s41598-021-84051-w

**Published:** 2021-03-02

**Authors:** Giulia Collodel, Fabio Ferretti, Maurizio Masini, Giacomo Gualtieri, Elena Moretti

**Affiliations:** 1 Department of Molecular and Developmental Medicine, University of Siena, Policlinico Le Scotte, Viale Bracci, 14, 53100 Siena, Italy; 2 Department of Medical and Surgical Sciences and Neurosciences, University of Siena, Siena, Italy; 3 Department of Social, Political and Cognitive Sciences, University of Siena, Siena, Italy; 4 Legal Medicine Unit, University Hospital, AOUS, Siena, Italy

**Keywords:** Risk factors, Electron microscopy, Urology, Urogenital diseases

## Abstract

The impact of male aging on male fertility has only recently become of interest to the scientific community. This study aims to assess the relationship between age and fertility among a sample of men, considering the individual and pathological characteristics. In this retrospective study data of semen analysis and medical history of 1294 Italian male patients were considered. Semen analysis was performed by light microscopy and transmission electron microscopy mathematically elaborated. A generalized linear model was used to explore the influence of male age on semen quality, considering as confounders wine consumption, smoking habits, presence of varicocele, consanguinity and positive semen bacteriological analysis and urethral swab. The mean age of the participants was 36.41 ± 6.379. Male aging without impact of confounders was correlated with a decrease in sperm concentration and motility and an increased in sperm necrosis. Sperm concentration and progressive motility were negatively related to the presence of confounders as wine consumption (sperm motility), urogenital infection (sperm concentration and motility), varicocele (sperm concentration) and consanguinity (sperm motility). Urogenital infection, varicocele and consanguinity positively correlated with sperm necrosis. The most important finding was the observation of a negative effect of male aging on sperm parameters such as concentration, motility, and viability. It is possible to hypothesize age-dependent changes of testicular environment, probably related to reactive oxygen species production. The demonstration, in a large sample of patients, that aging influences sperm quality strongly motivates further research focused on the mechanisms involved in this phenomenon and its effects on offspring fitness.

## Introduction

In the recent years, social pressures induce couples to delay pregnancies later in their life, into their mid-to late thirties. Extensive data on the negative effects of advanced maternal age on reproductive outcomes and fetal health are reported^1^. Increasing evidence indicates an age dependent decline also in sperm fitness^2^, this information is becoming important given the age at which men are fathering children has been increased over time^3,4^. The exact mechanisms, responsible for this reduction in sperm quality, are still not fully understood and the literature suggests various hypotheses. The decline in overall sperm quality with paternal age can be linked to the change in testicular function^5^, the damage by urological diseases^6^ and oxidative stress^7^. Studies on rats contributed to investigate the mechanisms of reproduction in aged males. It was reported that male reproductive aging is primarily a neuroendocrine dysfunction characterized by decreased pulsatile luteinizing hormone (LH) secretion leading to low serum testosterone levels, however, while in the Sprague–Dawley rat sperm production is relatively well maintained, in Brown-Norway decreased seminiferous tubule function is accompanied by elevated follicle stimulating hormone (FSH) levels indicative of a primary testicular disorder^8^.

The evaluation of male age has rekindled the interest of the potential impact that different factors as environmental factors, lifestyle, anatomical and inflammatory conditions may have on male fertility^9^. Until today there are conflicting data about the influence of the paternal age on seminal parameters^5,10,11^, also considering that study populations were often not clearly defined and fertile men are rarely used as controls^7^.

Light microscopy is the most common approach used to assess sperm parameters^12^, however, transmission electron microscopy (TEM) is the best method to perform a sperm pathology’s investigation and to characterize the ultrastructural sperm defects at different levels (acrosome, nucleus, chromatin, connecting piece, mitochondria, axonemal and periaxonemal structures)^13–15^.

Studies have also considered the possibility of age-related changes in sperm genetics, such as sperm DNA fragmentation^16–18^, sperm aneuploidy^19^, telomere length^20^ and epigenetics^21^. Moreover, an increased risk of congenital abnormalities, neuropsychiatric diseases^6^, autism^22^ and childhood acute lymphoblastic leukemia^23^ in offspring of men with advanced paternal age have been described.

Because the frequency of chromosomal anomalies in spermatozoa appears to increase with male age, an influence of paternal age on risk of spontaneous abortion has been suggested^24^. Increased paternal age was also related to a longer time to pregnancy^25^. Different groups have recently described adverse associations between male age and reproductive outcomes^26,27^. In addition, it is still debated if in vitro fertilization (IVF) and intracytoplasmic sperm injection (ICSI) may result to be negatively influenced by paternal age. By these techniques, a decline in fertilization, embryo quality, implantation, pregnancy, live birth rates and pregnancy loss have been observed^19,28–30^.

In this paper, we considered sperm parameters in a large cohort of men attending our laboratory for seminal analyses. Semen analysis was performed by light microscopy and transmission electron microscopy (TEM) mathematically elaborated. The obtained data were processed by a Generalized Linear Model that was fitted for each continuous outcome with the aim of analyzing the relationship between age and the semen parameters, controlling for the effects of other parameters as wine consumption, smoking habits, presence of varicocele, consanguinity and positive semen bacteriological analysis and urethral swab.

## Materials and methods

### Patients and inclusion criteria

In this retrospective study, we reviewed the semen analysis database of 1294 Italian male patients examined from January 1999 to October 2017 in our laboratory. The primary reason to seek counselling in our Centre was primary infertility defined as 2 years of unprotected sexual intercourse without conception, however we analysed also semen samples from men who want to check their fertility status, before undertake varicocele surgery and because testicular pain. We extracted demographic (age, occupation, height, weight, smoking, drinking history) and clinical information, their family background, and their possible consanguinity history. Routine checking provided for the level of testosterone, cortisol, estradiol, FSH, LH, prolactin, TSH, T3 and T4 in blood. The semen and urethral fluid were tested for asymptomatic genitourinary infection: a microbiological analysis was performed in semen samples and urethral swabs of all the patients. Patients showing positive bacteriological cultures were considered as infected.

The volume, position and consistency of the testes and epididymis were checked by a physical examination, each spermatic cord was palpated in the standing position and during the Valsalva maneuver and scrotal Eco-colour Doppler performed in all patients. Varicocele was classified in different grades and in right, left o bilateral. Patients with a subclinical varicocele were included in the study. Karyotype was also performed in all patients and in patients with a reduced number of sperm (< 10 × 10^6^sperm/ml) Y microdeletion testing was performed.

Inclusion criteria: complete information in the records, non azoospermic patients with a normal 46, XY karyotype, a normal hormonal profile, no history of radiotherapy, chemotherapy, chronic illness or medication, testicular cancer, drug consumption. None of the patients showed sperm defects of supposed genetic origin, characterized by an identical and specific alteration affecting most of the sperm population.

In addition, paraplegic and obese men (BMI > 25), men with occupational exposure to chemicals or excessive heat as well as carriers of altered karyotype and Y chromosome microdeletions were excluded.

At the time of the analysis, patients provided written informed consent for the inclusion in Centre’s research according to the guidelines of the period for respecting privacy and the Helsinki Declaration of 1975.

### Semen analyses

#### Light microscopy

Semen samples were collected by masturbation after days (3–5) of sexual abstinence and examined after liquefaction for 30 min at 37 °C. Patients were asked to urinate and wash the hands, penis and scrotum before ejaculation. Before the evaluation, an aliquot of each sample was recovered and sent in the laboratory for microbiological analysis. Volume, pH, sperm concentration and motility were assessed as recommended by World Health Organization guidelines^12,31^. In the cases attended our centre before 2010 the sperm motility was evaluated as rapid and slow (a + b)^31^, in the cases after 2010 as sperm progressive motility (recommended by the WHO guidelines^12^). Sperm morphology evaluated by light microscope has not been considered as it has been deeply investigated by TEM.

#### Transmission electron microscopy

For the TEM procedure, sperm samples were fixed in cold Karnovsky fixative and maintained at 4 °C for 2 h. Then the semen was washed in 0.1 mol/l cacodylate buffer (pH 7.2) for 12 h, postfixed in 1% buffered osmium tetroxide for 1 h at 4 °C and washed again in 0.1 mol/l cacodylate buffer. The samples were dehydrated in a graded ethanol series and embedded in Epon Araldite^32^. Ultra-thin sections were cut with a Supernova ultramicrotome (Reickert Jung, Vienna, Austria), mounted on copper grids, stained with uranyl acetate and lead citrate and then observed and photographed with a Philips CM10 and Philips CM12 transmission electron microscopes (TEM; Philips Scientifics, Eindhoven, The Netherlands, University of Siena and Centro di Microscopie Elettroniche “Laura Bonzi”, ICCOM, Consiglio Nazionale delle Ricerche –CNR-,Via Madonna del Piano,10 Firenze, Italy).

**Figure 1:**
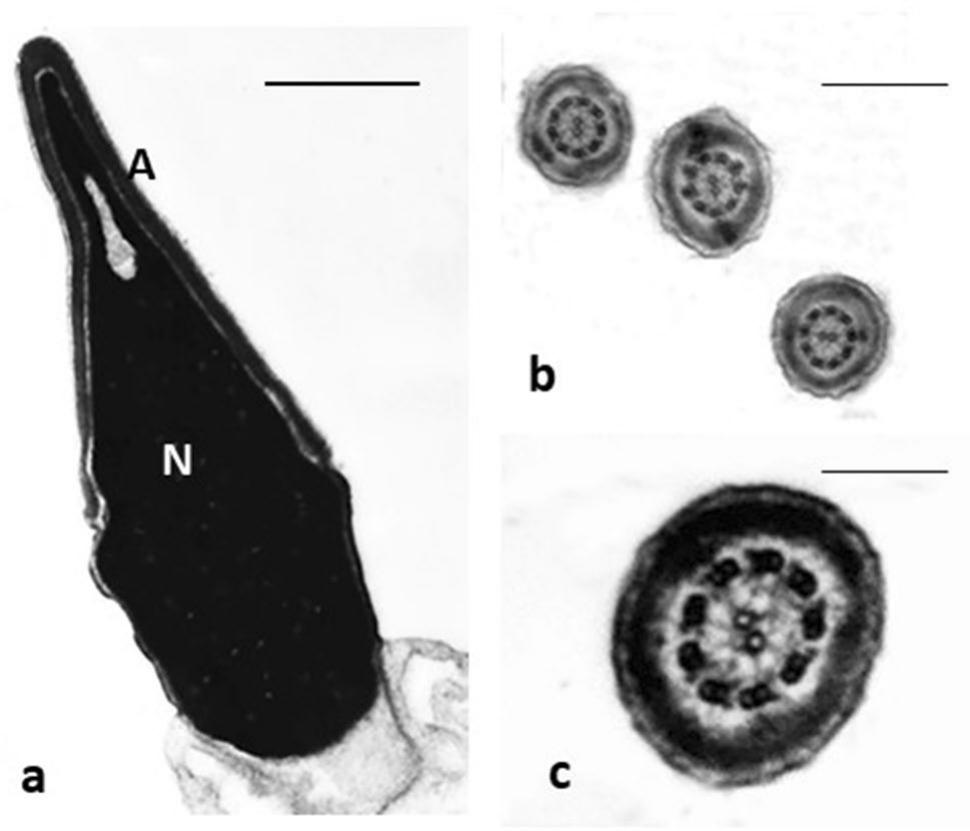
TEM micrographs of normal sperm. (**a**) longitudinal section of a sperm head: the nuclear shape is regular, and the chromatin condensed; (**b**,**c**) cross sections of tails with normal axonemal pattern. Nucleus (N), acrosome (A). Bars: (**a**) 1 µm; (**b**) 4 µm; (**c**) 2 µm.

It is well known that statistics obtained by TEM examination of ultrathin sections are imperfect and questionable; for this reason, a mathematical method to approach the problem of the sperm quality evaluation was proposed^32^ and used for more than 25 years in our laboratory. Proceeding with a Bayesian technique, we have developed a formula considering all the statistical possibilities for defects to be present in a sperm cell, the total number of affected spermatozoa, and, therefore, the sperm devoid of defects (Fig. 1). The ultrastructural defects analysed are referred to the acrosome (position, dimension, shape and content), the nucleus (normal shape, roundish shape, and altered shape), the chromatin texture (condensed, immature, necrotic and with holes), the centrioles, the mitochondria (shape and assembly), the axonemal (9 + 2 organization, presence of dynein arms) and periaxonemal structures (outer dense fibers and fibrous sheath), the plasma membrane (integer, broken) and the presence/absence of cytoplasmic residue.

**Figure 2:**
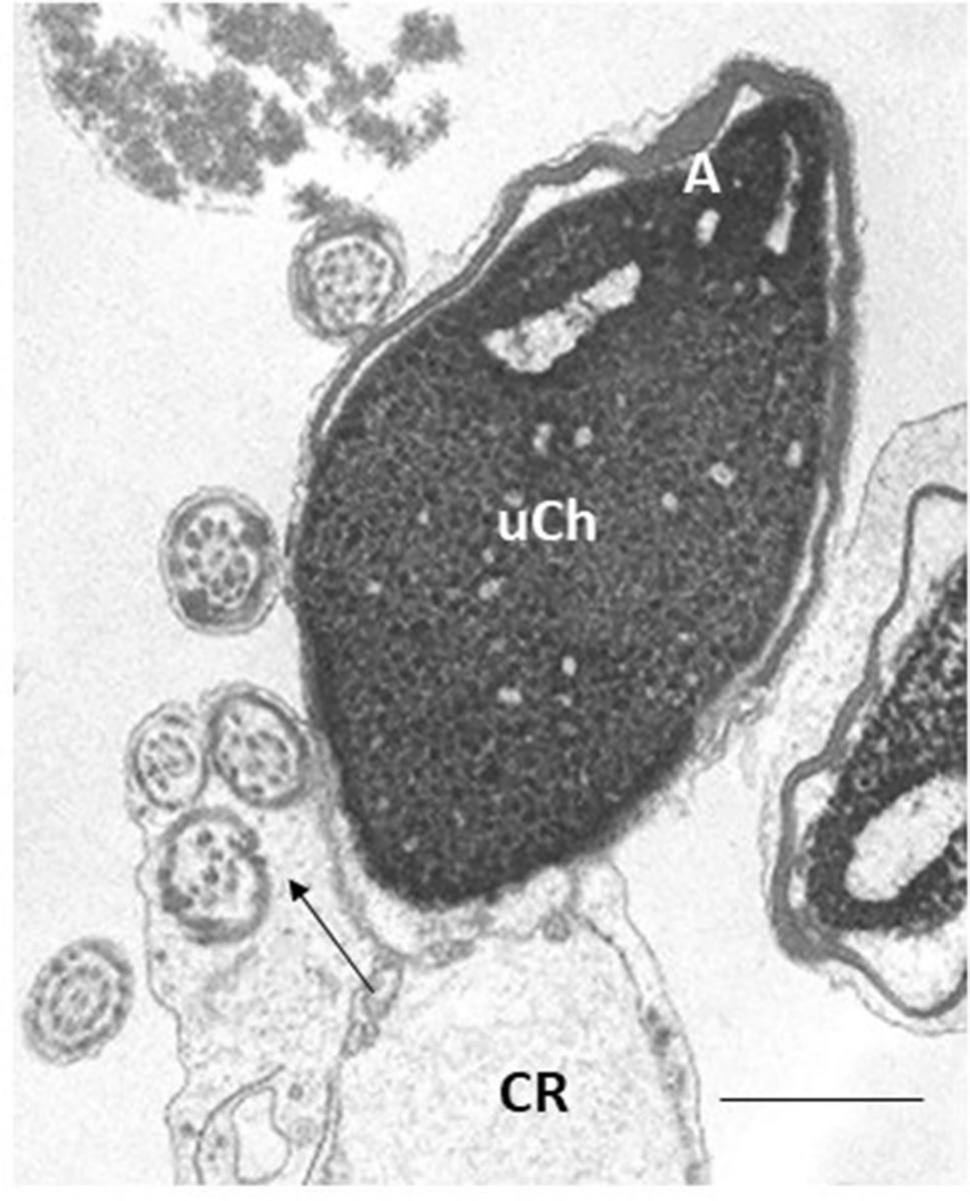
TEM micrograph of longitudinal section of an immature sperm. The acrosome (A) is anomalous in shape and the altered nucleus shows uncondensed chromatin (uCh). A cytoplasmic residue (CR) embedding the coiled tail with altered axonemal and periaxonemal structures (arrow) is present. Bar: 1 µm.

**Figure 3:**
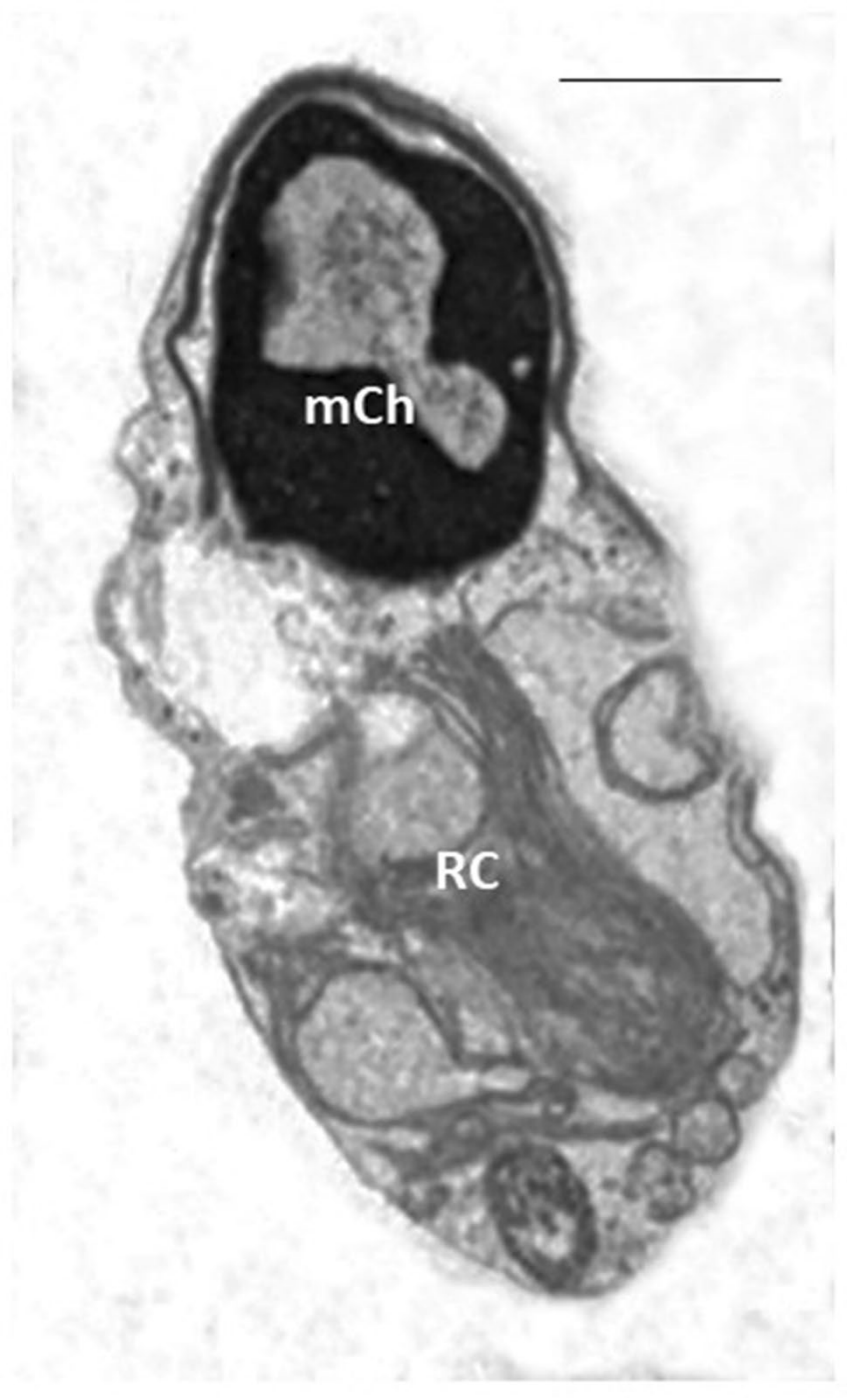
TEM micrograph of a longitudinal section of an apoptotic sperm showing altered nucleus with marginated chromatin (mCh) and the presence of a wide hole, a typical morphological characteristic of apoptosis. The acrosome does not completely fit with nuclear shape, a cytoplasmic residue (CR) and a section of an altered axonemal pattern are visible. Bar: 2 µm.

**Figure 4:**
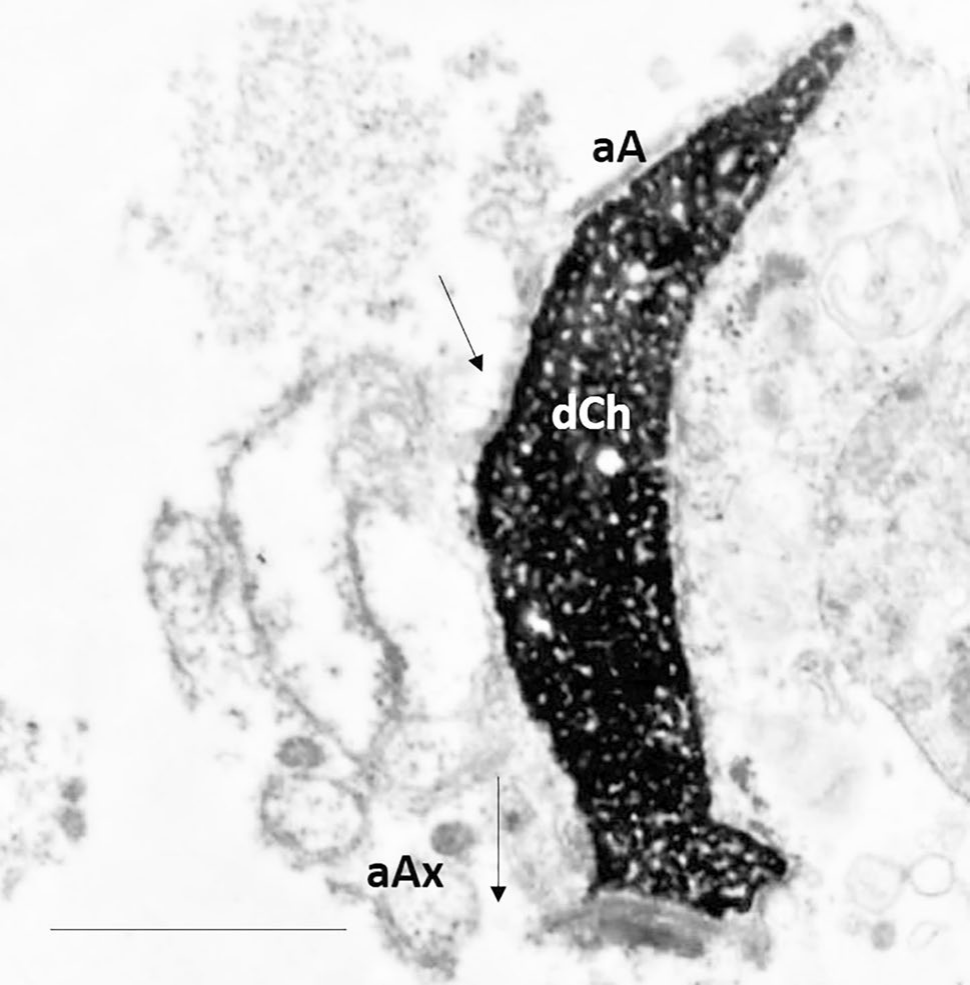
TEM micrograph of longitudinal section of necrotic sperm characterized by disrupted chromatin (dCh), acrosome with poor content (aA), broken plasma membrane (arrows) and altered axoneme (aAx). Bar: 2 µm.

Three hundred longitudinal and cross sperm sections, depending on the organelle or structure in analysis, were examined in each sample. The obtained data were uploaded in the mathematical formula that provides numerical scores such as fertility index (number of sperm free of structural defects in a semen sample) and the percentage of sperm pathologies such as immaturity, apoptosis and necrosis^33^. The typical traits of sperm immaturity include the presence of cytoplasmic droplets, altered acrosomes, roundish or elliptical nuclei with uncondensed chromatin (Fig. 2). Marginated chromatin, translucent vacuoles embedded in cytoplasmic residues, swollen and badly assembled mitochondria are the ultrastructural indicators of apoptosis (Fig. 3). Sperm with reacted or absent acrosome, misshaped nuclei with disrupted chromatin, broken plasma membrane and poor axonemal and periaxonemal cytoskeletal structures are affected by necrosis (Fig. 4).

### Statistical analysis

Descriptive statistics were used to summarize the main characteristics of the participants. Mean, standard deviation, and the 95% confidence interval for the mean were used to estimate the continuous outcomes of the study: semen volume (ml), semen pH, sperm concentration expressed as sperm/ml × 10^6^, progressive sperm motility, fertility index, sperm apoptosis, immaturity and necrosis.

According to Hoaglin and Iglewicz^34^ values of the continuous variables higher than 2.2 IQR (interquartile range)’s were considered outliers and excluded from the analysis, then the normal distribution of these measures was assessed by the Kolmogorov–Smirnov test.

A Generalized Linear Model was fitted for each continuous outcome with the aim of explaining the semen parameters (SV = semen volume, C = concentration, sperm/ml × 10^6^, SM = progressive sperm motility, SpH = semen pH, FI = fertility index, AP = sperm apoptosis, IM = sperm immaturity, NE = sperm necrosis) with age (AGE) as the predictor and controlling for main effects of the following categorical predictors: wine consumption (WC), smoking habit (SH), semen bacteriological analysis (BA), urethral swab (US), varicocele (VA) and consanguinity (CO). Each model was checked for basic assumptions (linearity, normality of residuals, and homoscedasticity). Multicollinearity was assessed comparing Type I and Type III Sum of Squares estimations of effects. The significance of the Omnibus test was checked to assess the predictive power of the models, whilst the goodness of fit was evaluated using the ratio between the deviance and the degrees of freedom, assuming that values lower than 2.5 provided an acceptable model. Due to the skewed distributions of the response variables, the models were fitted according to the Gamma distribution with a link function set up to identity. The model fitting, for example according to the following general form explaining the semen volume (SV), was performed for each outcome measure, but only those results with a significant relationship between AGE and the dependent variable were shown in this paper.

$$ SV = {b_0} + {b_1}\,WC + {b_2}\,SH + {b_3}\,BA + {b_4}\,US + {b_5}\,VA + {b_6}\,CO + {b_7}\,AGE + E$$

Interaction terms between AGE and the other predictors were tested only if AGE and the other independent variables were both significant. A post hoc power analysis was executed: given a sample size of 1294 participants, an effect size equal to 0.02, a value of α set up at 0.05, and 7 predictors, the determined power was 0.095. The statistical analyses were performed with the SPSS-IBM v. 25 software, and the level of significance was set at p < 0.05.

### Statement of human and animal rights

All procedures and the research protocol have been approved by the locally appointed ethics committee of the University of Siena. The experimental protocol was designed in accordance with the Declaration of Helsinki (1964).

### Informed consent

Informed consent was obtained from all individual participants included in the study. All authors were involved in study concept/study design or data acquisition, manuscript drafting or manuscript revision for important intellectual content, and manuscript final version approval.

## Results

**Figure 5:**
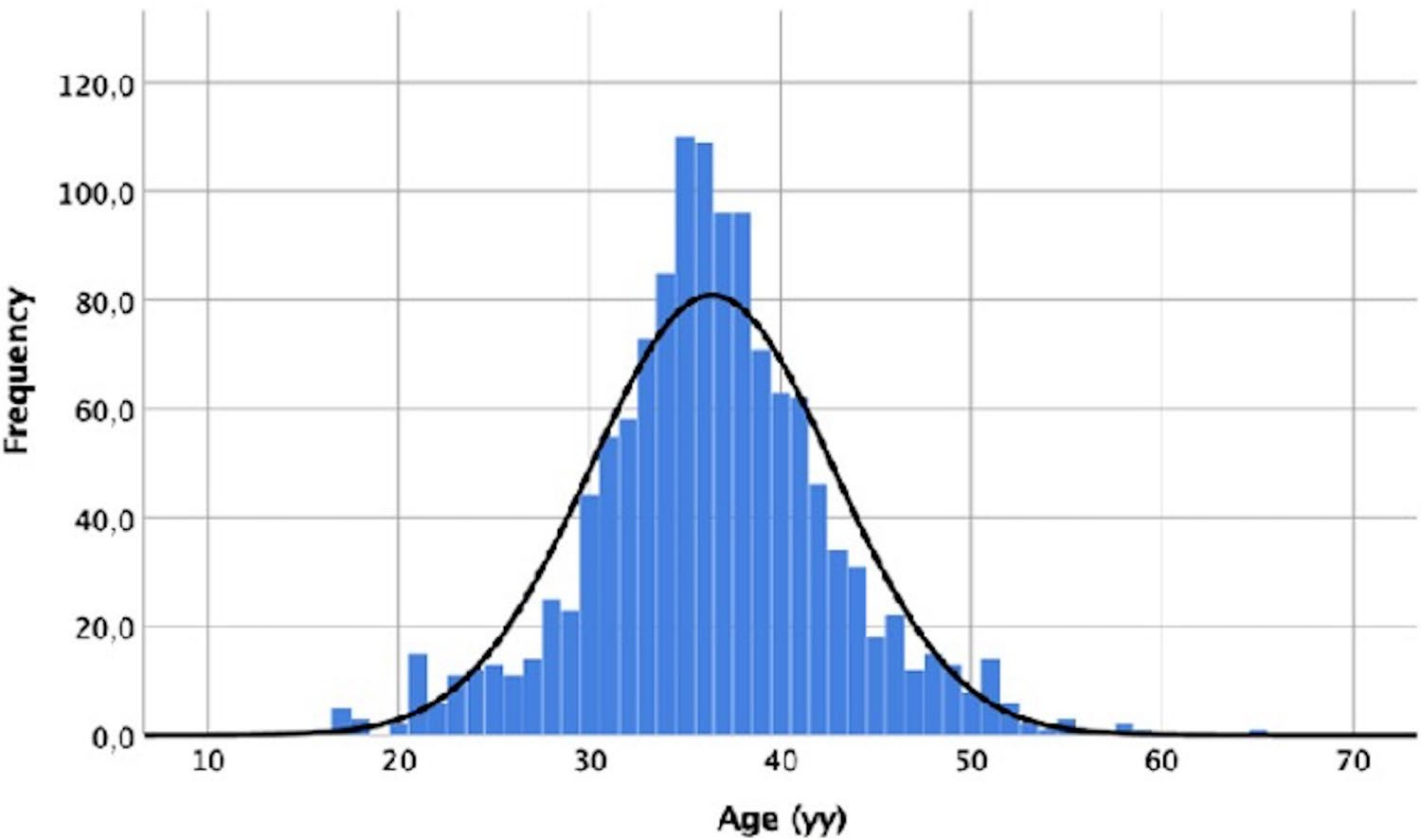
Graphic showing the distribution of age in the analysed sample (min = 16, max = 65; 95% CI 36.06–36.75).

In our study, a sample of 1294 men was considered. The mean age of the participants was 36.41 ± 6.379 (min = 16, max = 65; 95% CI 36.06–36.75, Fig. 5).

A large number of participants were not wine-drinkers (74.3%) and non-smokers (62.7%; Table 1).

**Table 1:** Participants characteristics (n = 1294).

Characteristics		n (%)
Wine consumption (WC)	No	961 (74.3)
	Less than 1 L/day	284 (21.9)
	1 L/day and more	49 (3.8)
Smoking habit (SH)	Not smoker	811 (62.7)
	1–9 cigarettes/day	108 (8.3)
	10 and more cigarettes/day	375 (29.0)
Semen bacteriological analysis (BA)	Negative	1061 (82.0)
	Positive	233 (18.0)
Urethral swab (US)	Negative	1138 (87.9)
	Positive	156 (12.1)
Hydrocele	No	1246 (96.3)
	Yes	48 (3.7)
Varicocele (VA)	No	822 (63.5)
	Yes	472 (36.5)
Cryptorchidism	No	1237 (95.6)
	Yes	57 (4.4)
Testicular volume	Normal range	1177 (91.0)
	Out of the normal range	117 (9.0)
Testicle lift	No	1262 (97.5)
	Yes	32 (3.2)
Inguinal hernia	No	1275 (98.5)
	Yes	19 (1.5)
Consanguinity (CO)	No	1250 (96.6)
	Yes	44 (3.4)

Eighty-two % of participants showed positive BA and 12.1% positive US. Testicular volume was out of normal range in 9% of patients. VA was the most frequent observed pathology: 36.5% of the subjects was affected, a high percentage if compared with other pathologies, such as hydrocele (3.7%), cryptorchidism (4.4%), testicle lift (3.2%) and inguinal hernia (1.5%). The condition of CO affected 3.4% of the patients (Table 1).

The seminological features of the studied patients were analysed by light and transmission electron microscopies and means ± standard deviations were reported in Table 2. Volume, pH, sperm concentration and progressive motility data referred to WHO^12^ values, fertility index, sperm apoptosis, immaturity and necrosis percentages were compared to reference values reported in Collodel and Moretti^33^. Our sample highlighted a reduced progressive motility and an increased percentage in sperm apoptosis, immaturity, and necrosis.

**Table 2:** Semen parameters of the patients’ sample (n = 1294) and the inferential estimations of these measures (95% CI).

Semen parameters	Mean ± SD	SE	95% CI Lower limit Upper limit		Reference values
Volume (ml)	3.480 ± 1.735	0.048	3.385	3.575	25th percentile*
pH	7.678 ± 2.723	0.076	7.529	7.827	≥ 7,2*
Sperm/ml × 10^6^	85.080 ± 107.789	3.001	79.193	90.968	50th percentile*
Sperm progressive motility %	29.438 ± 20.988	0.584	28.292	30.584	< 5th percentile*
Fertility index	3,838,491.383 ± 14,797,996.282	412,010.286	3,030,207.098	4,646,775.668	7,386,079.7 ± 10,464,288**
Sperm apoptosis %	9.312 ± 7.437	0.207	8.906	9.718	4.056 ± 2.048**
Sperm immaturity %	66.377 ± 14.384	0.400	65.591	67.162	48.83 ± 13.93**
Sperm necrosis %	40.342 ± 18.040	0.502	39.357	41.328	32.13 ± 10.58**

Sperm characteristics were evaluated by light microscopy (sperm/ml × 10^6^, sperm progressive motility) and by TEM (fertility index, sperm apoptosis, immaturity, necrosis). *^12^; **^33^.

The results of the generalized linear models used to analyse the relationship between the AGE and the semen parameters, controlling for a set of individual conditions related to lifestyle and presence of pathologies, are shown in the next paragraphs.

### Semen volume (SV)

Although satisfactory goodness of fit was found (deviance/degrees of freedom(df) = 0.255), the Omnibus test failed to reject the null hypothesis: all slope parameters are not significantly different from zero, therefore there was no evidence of a significant contribution of the predictors to the values of the SV. No multicollinearity was detected and none of the independent variables showed a significant parameter.

### Semen pH (SpH)

The model estimated with SpH as a response variable revealed a very good fit (deviance/df = 0.021), with the rejection of the Omnibus test null hypothesis, but AGE didn’t provide a significant characteristic (Table 3). The only significant main effect was observed for VA: the patients who were not affected by this testicular pathology presented a higher pH value [*β* = − 0.263, Wald *χ*^*2*^_(1)_ = 15.649, p = 0.000]. No multicollinearity was found between the predictors.

**Table 3:** Results of the generalized linear model with semen pH (SpH) as response variable.

Characteristics	β	Standard Error	Wald's Chi-square	p
Intercept	7.629	0.314	χ^2^_(1)_ = 574.549	0.000
WC—No	0.081	0.166	χ^2^_(1)_ = 0.239	0.625
WC—Less than 1 L/day	− 0.064	0.174	χ^2^_(1)_ = 0.134	0.175
WC—1 L/day and more	0.00	–	–	–
SH—Not smoker	0.105	0.071	χ^2^_(1)_ = 2.207	0.137
SH—1–9 cigarettes/day	− 0.040	0.122	χ^2^_(1)_ = 0.105	0.746
SH—10 and more cigarettes/day	0.00	–	–	–
BA—Negative	0.131	0.089	χ^2^_(1)_ = 2.164	0.141
BA—Positive	0.00	–	–	–
US—Negative	0.065	0.105	χ^2^_(1)_ = 0.385	0.535
US—Positive	0.00	–	–	–
VA—No	− 0.263	0.067	χ^2^_(1)_ = 15.649	0.000
VA—Yes	0.00	–	–	–
CO—No	0.018	0.174	χ^2^_(1)_ = 0.011	0.916
CO—Yes	0.00	–	–	–
AGE	− 0.002	0.005	χ^2^_(1)_ = 0.118	0.732

*WC* wine consumption, *SH* smoking habit, *BA* semen bacteriological analysis, *US* urethral swab, *VA* varicocele, *CO* consanguinity.

### Sperm concentration (1 × 10^6^/ml, C)

The results of the generalized linear model with C as the response variable are presented in Table 4. A main effect of AGE was found on levels of C: controlling for the other predictors, older participants reported a reduced C [*β* = − 0.816, Wald *χ*^*2*^_(1)_ = 4.206, p = 0.040]. A significant main effect was also detected for BA, US and VA. Patients with negative BA provided higher values of C than subjects with positive BA [*β*  = 16.263, Wald *χ*^*2*^_(1)_ = 4.108, p = 0.043], patients with negative US showed an increased C as well [*β* = 25.428, Wald χ^2^_(1)_ = 12.099, p = 0.001]; the absence of VA was associated with an increased C [*β* = 16.087, Wald χ^2^_(1)_ = 7.837, p = 0.005]. The estimation of the interaction terms of these three variables with AGE did not provide any significant result. The model showed excellent goodness of fit (deviance/df = 1.536), and the null hypothesis of the parameters’ slope equal to zero was rejected by the Omnibus test (p = 0.000). No multicollinearity between predictors was detected.

**Table 4:** Results of the generalized linear model with sperm/ml × 10^6^ (C) as response variable.

Characteristics	β	Standard error	Wald's Chi-square	P
Intercept	62.032	25.684	χ^2^_(1)_ = 5.833	0.016
WC—No	9.983	11.023	χ^2^_(1)_ = 0.802	0.365
WC—Less than 1 L/day	11.502	11.945	χ^2^_(1)_ = 0.927	0.336
WC—1 L/day and more	0.00	–	–	–
SH—Not smoker	11.008	5.783	χ^2^_(1)_ = 3.623	0.057
SH—1–9 cigarettes/day	19.721	14.982	χ^2^_(1)_ = 1.733	0.188
SH—10 and more cigarettes/day	0.00	–	–	–
BA—Negative	16.263	8.024	χ^2^_(1)_ = 4.108	0.043
BA—Positive	0.00	–	–	–
US—Negative	25.428	7.310	χ^2^_(1)_ = 12.099	0.001
US—Positive	0.00	–	–	–
VA—No	16.087	5.747	χ^2^_(1)_ = 7.837	0.005
VA—Yes	0.00	–	–	–
CO—No	15.025	12.702	χ^2^_(1)_ = 1.399	0.237
CO—Yes	0.00	–	–	–
Age	− 0.816	0.043	χ^2^_(1)_ = 4.206	0.040

*WC* wine consumption, *SH* smoking habit, *BA* semen bacteriological analysis, *US* urethral swab, *VA* varicocele, *CO* consanguinity.

### Sperm progressive motility (SM)

Considering SM as a response variable (Table 5), AGE represented a significant characteristic: an increasing age was correlated to a decreasing SM [*β* = − 0.232, Wald *χ*^*2*^_(1)_ = 7.376, p = 0.007], controlling for the other predictors. Among the independent variables, WC, BA and CO gave a significant contribution to the values of SM, revealing a negative impact on SM. A low or absent WC seems to play a positive influence on SM [no consumption: *β* = 5.979, Wald *χ*^*2*^_(1)_ = 6.175, p = 0.013; less than 1 L/day: *β*  = 5.978, Wald *χ*^*2*^_(1)_ = 5.345, p = 0.021], a negative BA predicted an increased SM [*β* = 5.494, Wald χ^2^_(1)_ = 13.400, p = 0.000] and finally the absence of CO was associated to an improvement of this sperm parameter [*β* = 8.082, Wald χ^2^_(1)_ = 11.669, p = 0.001]. Once again, the estimation of the interaction terms of these three variables with AGE did not provide any significant results. The model showed excellent goodness of fit (deviance/df = 0.474), and the null hypothesis of the parameters’ slope equal to zero was rejected by the Omnibus test (p = 0.000). No multicollinearity between predictors was found.

**Table 5:** Results of the generalized linear model with sperm progressive motility (SM) as response variable.

Characteristics	β	Standard Error	Wald's Chi-square	p
Intercept	20.374	4.991	χ^2^_(1)_ = 16.664	0.000
WC—No	5.979	2.406	χ^2^_(1)_ = 6.175	0.013
WC—Less than 1 L/day	5.978	2.586	χ^2^_(1)_ = 5.345	0.021
WC—1 L/day and more	0.00	–	–	–
SH—Not smoker	0.533	1.253	χ^2^_(1)_ = 0.181	0.670
SH—1–9 cigarettes/day	− 0.892	2.174	χ^2^_(1)_ = 0.168	0.682
SH—10 and more cigarettes/day	0.00	–	–	–
BA—Negative	5.494	1.501	χ^2^_(1)_ = 13.400	0.000
BA—Positive	0.00	–	–	–
US—Negative	− 1.475	1.844	χ^2^_(1)_ = 0.640	0.424
US—Positive	0.00	–	–	–
VA—No	0.927	1.189	χ^2^_(1)_ = 0.607	0.436
VA—Yes	0.00	–	–	–
CO—No	8.082	2.366	χ^2^_(1)_ = 11.669	0.001
CO—Yes	0.00	–	–	–
AGE	− 0.232	0.085	χ^2^_(1)_ = 7.376	0.007

*WC* wine consumption, *SH* smoking habit, *BA* semen bacteriological analysis, *US* urethral swab, *VA* varicocele, *CO* consanguinity.

### Fertility index (FI)

Due to the poor fitting of the model with FI as a response variable, the results of the estimations were ignored. The value of the ratio between the deviance and the degrees of freedom was 18.853, largely above the threshold that was considered as a criterion for the goodness of fit assessment (2.5).

### Sperm apoptosis (AP)

The generalized linear model estimated for AP (Fig. 3) showed an excellent fit (deviance/df = 0.767), rejection of the null hypothesis of all slope parameters equal to zero, but with a not significant AGE’s characteristic (Table 6). BA, VA and CO displayed a significant contribution to the AP determination. In particular, subjects with negative BA were characterized by lower values of AP [*β* = − 1.458, Wald *χ*^*2*^_(1)_ = 4.824, p = 0.028] and the same effect was observed in association with the absence of testicular pathologies as VA [*β* = − 1.027, Wald *χ*^*2*^_(1)_ = 4.972, p = 0.026] and with the absence of CO [*β* = − 3.783, Wald *χ*^*2*^_(1)_ = 5.270, p = 0.022]. No multicollinearity was detected among the independent variables.

**Table 6:** Results of the generalized linear model with sperm apoptosis (AP) as response variable.

Characteristics	β	Standard Error	Wald's Chi-square	p
Intercept	15.467	2.452	χ^2^_(1)_ = 39.785	0.000
WC—No	0.984	1.037	χ^2^_(1)_ = 0.901	0.342
WC—Less than 1 L/day	0.948	1.109	χ^2^_(1)_ = 0.732	0.392
WC—1 L/day and more	0.00	–	–	–
SH—Not smoker	− 0.696	0.496	χ^2^_(1)_ = 1.972	0.160
SH—1–9 cigarettes/day	− 0.583	0.851	χ^2^_(1)_ = 0.469	0.493
SH—10 and more cigarettes/day	0.00	–	–	–
BA—Negative	− 1.458	0.664	χ^2^_(1)_ = 4.824	0.028
BA—Positive	0.00	–	–	–
US—Negative	0.378	0.727	χ^2^_(1)_ = 0.271	0.603
US—Positive	0.00	–	–	–
VA—No	− 1.027	0.461	χ^2^_(1)_ = 4.972	0.026
VA—Yes	0.00	–	–	–
CO—No	− 3.783	1.648	χ^2^_(1)_ = 5.270	0.022
CO—Yes	0.00	–	–	–
AGE	− 0.040	0.037	χ^2^_(1)_ = 1.145	0.285

*WC* wine consumption, *SH* smoking habit, *BA* semen bacteriological analysis, *US* urethral swab, *VA* varicocele, *CO* consanguinity.

### Sperm immaturity (IM)

Considering the model with IM (Fig. 2) as a dependent variable (Table 7) and controlling for the other predictors, the variable AGE highlighted a borderline significance and a week evidence that an increasing age was correlated to an decreasing value of IM [*β* = − 0.134, Wald *χ*^*2*^_(1)_ = 3.751, p = 0.053]. Two predictors provided a significant contribution to IM: BA and VA. Negative BA was predictive of a lower level of IM [*β* = − 2.949, Wald *χ*^*2*^_(1)_ = 5.571, p = 0.018] as well as the absence of VA was associated with a lower IM [*β* = − 2.974, Wald *χ*^*2*^_(1)_ = 10.883, p = 0.001]. The goodness of fit is very satisfactory (deviance/df = 0.053), and the null hypothesis of the parameters’ slope equal to zero was rejected by the Omnibus test (p = 0.002). No multicollinearity was detected.

**Table 7:** Results of the generalized linear model with sperm immaturity (IM) as response variable.

Characteristics	β	Standard Error	Wald's Chi-square	p
Intercept	76.012	4.340	χ^2^_(1)_ = 306.125	0.000
WC—No	− 0.283	2.265	χ^2^_(1)_ = 0.016	0.901
WC—Less than 1 L/day	− 1.040	2.378	χ^2^_(1)_ = 0.191	0.662
WC—1 L/day and more	0.00	–	–	–
SH—Not smoker	− 0.536	0.971	χ^2^_(1)_ = 0.305	0.581
SH—1–9 cigarettes/day	− 2.525	1.646	χ^2^_(1)_ = 2.353	0.125
SH—10 and more cigarettes/day	0.00	–	–	–
BA—Negative	− 2.949	1.249	χ^2^_(1)_ = 5.571	0.018
BA—Positive	0.00	–	–	–
US—Negative	1.835	1.436	χ^2^_(1)_ = 1.633	0.201
US—Positive	0.00	–	–	–
VA—No	− 2.974	0.902	χ^2^_(1)_ = 10.883	0.001
VA—Yes	0.00	–	–	–
CO—No	− 1.132	2.399	χ^2^_(1)_ = 0.223	0.637
CO—Yes	0.00	–	–	–
AGE	− 0.134	0.0691	χ^2^_(1)_ = 3.751	0.053

*WC* wine consumption, *SH* smoking habit, *BA* semen bacteriological analysis, *US* urethral swab, *VA* varicocele, *CO* consanguinity.

### Sperm necrosis (NE)

The final model was estimated with NE (Fig. 4) as the response variable (Fig. 2, Table 8). AGE was found significant: controlling for the other predictors, older participants reported a higher level of NE [*β*  = 0.424, Wald *χ*^*2*^_(1)_ = 32.067, p = 0.000]. A significant main effect was also detected for BA, VA and CO: patients with negative BA presented a lower value of NE [*β* = − 4–197, Wald *χ*^*2*^_(1)_ = 8.104, p = 0.005], the same effect was observed in association with the absence of VA [*β* = − 2.450, Wald *χ*^*2*^_(1)_ = 5.510, p = 0.019] and with the absence of CO [*β* = − 7.890, Wald *χ*^*2*^_(1)_ = 5.673, p = 0.017]. The estimation of the interaction terms of these three variables with AGE did not provide any significant results. The model showed excellent goodness of fit (deviance/df = 0.222), and the null hypothesis of the parameters’ slope equal to zero was rejected by the Omnibus test (p = 0.000). No multicollinearity between predictors was detected.

**Table 8:** Results of the generalized linear model with necrosis (NE) as response variable.

Characteristics	β	Standard Error	Wald's Chi-square	p
Intercept	42.219	5.068	χ^2^_(1)_ = 69.398	0.000
WC—No	− 3.086	2.359	χ^2^_(1)_ = 1.711	0.191
WC—Less than 1 L/day	− 3.812	2.512	χ^2^_(1)_ = 2.302	0.129
WC—1 L/day and more	0.00	–	–	–
SH—Not smoker	− 0.217	1.106	χ^2^_(1)_ = 0.038	0.845
SH—1–9 cigarettes/day	1.793	1.894	χ^2^_(1)_ = 0.896	0.344
SH—10 and more cigarettes/day	0.00	–	–	–
BA—Negative	− 4.197	1.483	χ^2^_(1)_ = 8.014	0.005
BA—Positive	0.00	–	–	–
US—Negative	− 1.718	1.785	χ^2^_(1)_ = 0.926	0.336
US—Positive	0.00	–	–	–
VA—No	− 2.450	1.044	χ^2^_(1)_ = 5.510	0.019
VA—Yes	0.00	–	–	–
CO—No	− 7.890	3.313	χ^2^_(1)_ = 5.673	0.017
CO—Yes	0.00	–	–	–
AGE	0.424	0.075	χ^2^_(1)_ = 32.067	0.000

*WC* wine consumption, *SH* smoking habit, *BA* semen bacteriological analysis, *US* urethral swab, *VA* varicocele, *CO* consanguinity.

## Discussion

In this research, a generalized linear model was used to analyse the effect of factor age on sperm characteristics excluding the impact of some confounders such as wine consumption, smoking habit, presence of genitourinary infections, varicocele, and consanguinity. For this reason, any conclusion drawn on this effect was controlled from interference due to the presence of these negative conditions related to male infertility. In literature, the role of confounders is not always considered^35,36^ and when it is considered the methodological approaches are very different as well as the variables considered as confounders. The limit to make comparisons that are blurred by regional variations, methodological bias and interpersonal variability found in semen from men^11^ is evident. For example, in a meta-analysis study that considers 90 papers, sample source, mean age, gross domestic product for countries involved in the study and abstinence were used as confounder variables^2^. Duration of abstinence, smoking, parity and other confounders were considered in the review of Kidd et al.^10^. A negative correlation between age and routine semen parameters was described also by Veron et al.^37^. In addition, they compared semen parameters in a selected subpopulation of aged men unexposed to known fertility-compromising factors as abnormally high BMI, alcohol consumption, cigarette smoking with those detected in older men not affected by these unhealthy conditions. They found that these factors played a paramount role in sperm quality deterioration^37^.

In this research statistical approach based on a Generalized Linear Model showed that in increasing paternal age, sperm concentration and motility decrease, and sperm necrosis grows, although the literature reports conflicting data related to the relationship between paternal age and sperm parameters.

The Gaussian distribution of patient’s age indicated that, although most of the cases settled in the rather narrow range of 30–36 years, the statistical method was sensitive enough to reveal the association between the variables considered and the paternal age.

Analyzing the single semen parameter, the semen volume is unrelated to aging as reported also by other authors^37^. However, the influence of the age on this parameter is not clear enough since the studied cohorts are extremely heterogeneous^2,37^.

The observed influence of aging on sperm concentration and motility was in accord with other literature reports. For example, Pasqualotto et al.^38^ identified an age threshold of > 45 years for sperm concentration and motility reduction. Stone et al.^35^ reported that sperm concentration declined after 40 years of age and sperm motility decreased after 43 years of age and, in addition, a decrease in total sperm count and sperm progressive motility were associated with advancing age^36^. In a meta-analysis review, Johnson et al.^2^ observed age-associated declines in semen volume, percentage motility, normal morphology and unfragmented cells, but not in sperm concentration.

The age-dependent influence in male reproductive organs such as testes and prostate were described and, consequently, variations in semen parameters over time are plausible^5^.

The relationship between paternal age and sperm morphology is difficult to interpret since the morphology criteria have been changed over the time and are variable between laboratories. In this study, sperm morphology was evaluated by TEM analysis mathematically elaborated for homogeneity of data since we included cases dating back 2010, date when the WHO updated the normal ranges for semen analysis. TEM method mathematically elaborated^32,33^ provides scores as fertility index, percentage of sperm apoptosis, immaturity, and necrosis. Applying the statistical method, the only score related to aging is sperm necrosis. Sperm necrosis is characterised by morphological features as disrupted chromatin, swollen mitochondria and broken plasma membrane, and the link between sperm necrosis and aging is in line with the increase of DNA fragmentation in advanced paternal age described by other authors^39^. Many studies reported a positive correlation between increasing male age and sperm DNA damage^40,41^ doubling from 25 to 55 years of age^42^. Kaarouch et al.^19^ studied spermatozoa of a group of aged men and found, despite normal sperm parameters, a significant increase in sperm DNA fragmentation, chromatin decondensation and sperm aneuploidy percentages compared to those detected in a group of young men. These alterations may suggest a link between male aging and changes in the testicular environment, particularly with the increase of reactive oxygen species production by mitochondria^43^. It is known that reactive oxygen species produced by mitochondria affect the integrity of the sperm genome and epigenome^44^ influencing both spermatogenesis and spermiogenesis processes^45,46^.

Recently, Garanina et al.^15^ studied the centrosomal region of two infertile patients and introduced the length of centriolar adjunct, as marker of sperm incomplete maturation that can affect fertility and might be responsible of the zygote arrest. The study of centriolar adjunct in human sperm is still little explored but it is worth of future investigations also in sperm of aged men.

The variable AGE is not correlated with sperm immaturity, apoptosis, and fertility index. Other authors^47^ found major associations between age and the frequencies of sperm with DNA fragmentation but not with sperm chromatin immaturity. Regarding sperm apoptosis, this data is in accord with some observations made in testes of old mice that showed low apoptotic frequencies than young adults^48,49^, however the correlation between aging and sperm DNA integrity represents another controversial question. Using different methods of investigations, it was suggested a detrimental effect of advanced paternal age on sperm chromatin integrity or DNA fragmentation^17,18,50,51^. TEM analysis enabled to clearly discriminate the chromatin status (uncondensed, disrupted and with holes) evaluating at same time different sperm characteristics as acrosome, plasma membrane, mitochondria, axoneme, and, after mathematical elaboration, it indicated that the chromatin damage was especially referred to necrosis more than apoptosis and immaturity. Moreover, ultrastructural characteristics do not always have a close relationship with molecular investigation that shows DNA fragmentation.

Fertility index is a score obtained by a mathematical elaboration of TEM data that expresses the number of sperm free of ultrastructural defects and strictly depends on the total number of spermatozoa of an ejaculate. Having considered a large heterogeneous population of men, this score has lost its relevance. Instead, fertility index assumes a key role in the comparisons between groups of patients with different pathologies or before and after a treatment^52^.

The applied statistical procedure also provided data on the effect that the single condition, considered as confounders for variable AGE, had on the semen quality. However, these conditions (WB, SH, BA, US, VA, CO) were not reciprocally controlled by the interference of the others but generally their influence on sperm parameters is in line with the data reported in literature.

Urogenital infections and varicocele negatively influenced sperm concentration and motility^53,54^ and increased sperm necrosis and apoptosis^52^. A high percentage of sperm immaturity was related to varicocele confirming its role as a typical marker of this pathology^55^.

Sperm progressive motility was also negatively influenced by wine consumption and presence of consanguinity, the last one a known condition for reduced motility^56–58^. The negative effect of varicocele on sperm progressive motility is known^59,60^, however, in this studied population, varicocele did not affect this sperm parameter. These contradictory results could be explained hypothesizing that the relationship between the presence of varicocele and sperm motility does not reach statistical significance since in the considered large population of patient’s subclinical varicocele was also included^61^. In addition, the selection criteria that considers both infertile patients and men who want to check their fertility status play an influence on the obtained results. Finally, it should be considered that, as mentioned above, the statistical method does not control this variable from the interference of the others, and therefore could be partially influenced by other factors.

Many studies on male aging were published and most of them revised results obtained in different laboratories. They were performed on men attending infertility clinics^10,62^ or on healthy non-smoking population^63,64^. Other authors have speculated that the association between decline in sperm quality and aging is not a direct effect of aging itself, but it is due to the influence of cumulative effects of lifelong exposures to toxins and pollutants^10^.

We believe that the main strengths of the present research are represented by the large sample of men attending the same laboratory over time, the information homogeneously collected and recorded in a single database, the evaluation of the different contributions of confounding factors as modulators.

Since the age of men included in the assisted reproduction programs is increasing, the knowledge on male age influence in sperm quality is of pivotal interest. At this purpose, Garcia-Ferreyra et al.^65^ recommend a genetic screening in embryo from egg donor cycles and in particular if paternal age is ≥ 50 years in order to obtain better clinical outcome and reduce the likelihood of abnormal pregnancies that may end in spontaneous abortions, intrauterine fetal death, intrauterine growth retardation or offspring with several congenital defects. It could be suggested that DNA fragmentation and sperm necrosis evaluation should be routinely screened for men of advanced age and patients advised of the potential risks. Then, clinicians should counsel old potential fathers on the risks of genetic diseases. Questionable, different ideas suggest young men to preserve their semen, probably creating additional ethical and financial concerns or recommend a limit in paternal age for assisted reproductive technologies, both proposals are highly controversial for numerous and obvious reasons^66^. Currently guidelines are not yet available^67^.

Curiously, old males of zebrafish have offspring with high fitness, despite declines in sperm performance and mating success, compensating benefits for declining fertility with age^68^. In humans, it has been hypothesized, but far from be proven, that the increasing telomere length in the sperm of older men may serve as a mechanism of “adaptive intergenerational plasticity” allowing for longer lifespan as generations reproduce at older ages^20^.

We hope our paper will stimulate a research that identifies the mechanisms underlying the age-based decline in sperm quality and performance, as well as those underlying the effects of male aging on offspring fitness.
